# Dependence of NMR noise line shapes on tuning, matching, and transmission line properties

**DOI:** 10.1002/cmr.b.21253

**Published:** 2014-02-24

**Authors:** Eli Bendet-Taicher, Norbert Müller, Alexej Jerschow

**Affiliations:** 1 Chemistry Department, New York UniversityNew York, NY, 10003; 2 Institute of Organic Chemistry, Johannes Kepler UniversityAltenbergerstraße 69, 4040, Linz, Austria

**Keywords:** NMR noise, transmission line

## Abstract

The tuning and matching conditions of rf circuits, as well as the properties of the transmission lines connecting these to the preamplifier, have direct consequences for NMR probe sensitivity and as for the optimum delivery of rf power to the sample. In addition, tuning/matching conditions influence radiation damping effects, which manifest themselves as fast signal flip-back and line broadening effects, and can lead to concentration-dependent frequency shifts. Previous studies have also shown that the appearance of spin-noise and absorbed circuit noise signals heavily depended on tuning settings. Consequently, all these phenomena are linked together. The mutual connections and interdependences of these effects are highlighted and reviewed here.

## INTRODUCTION

NMR probes are typically tuned to the desired resonance frequencies and matched to 50 Ω (if the characteristic impedance of the transmission line is also 50 Ω) at the frequency of interest. Using matched impedances assures the optimal transfer efficiency between probe and transmission line. The preamplifier input impedance, however, is often different from 50 Ω, but its noise characteristics are adjusted such that the highest signal-to-noise ratio (SNR) is obtained when the probe is matched to this value (as discussed in further detail below).

It has been known for a long time that radiation damping effects depend on tuning characteristics 1, the preamplifier input impedance, as well as on cable length. It was further seen that sizable radiation damping-induced frequency shifts 2–4 can occur, which change as a function of tuning frequency.

In recent spin-noise 5 experiments it was seen that there can exist a significant difference between the conventional tuning optimum (CTO) and the “on-resonance” condition for spin-noise line shapes, also called spin-noise tuning optimum (SNTO). In related experiments, it was found that the tuning conditions for optimal transmission vs. optimal reception could differ significantly for different NMR probes 2,6–8. We refer to spin-noise as the process of spontaneous emission of signals by the spin system, and absorbed circuit noise as the process of absorption of circuit noise by the spin system. The measurement of the interactions between the spins and the circuit Johnson–Nyquist noise allowed one to use these internal rf signal sources as indicators for the behavior of the received signals and probe properties 9. This approach was in many instances easier to implement than measuring the reception tuning sensitivity directly, especially for samples containing large signals (such as those from bulk H_2_O), where solvent suppression techniques are difficult to implement reproducibly over a range of tuning conditions. Examples of this tuning procedure have been shown in both ambient temperature and cryogenically cooled probes 6,7, as well as with hyperpolarized spins 10,11 and with solid-state NMR probes 7,8. In that work, the appearance of a symmetrical “dip” spin-noise line shape was seen as the condition for optimal detection. In several cases, however, it was observed that such a noise line shape was impossible to attain, or that the tuning conditions for a symmetric spin-noise dip (SNTO) and the conditions for optimal detection sensitivity were different. This discrepancy will also be discussed further below.

Starting from a description of spin-noise and absorbed circuit noise line shapes, we describe below the connections between tuning, frequency shifts, radiation damping 12–14, quality factors, and the observed noise line shapes. Most importantly, we discuss the influence of the cable length connecting the preamplifier and the probe circuit, and demonstrate the periodic effects of the parameters, in addition to the factors that influence optimal reception tuning, and tuning for symmetric spin-noise line shapes.

## THEORY

### Spin-Noise

Nuclear magnetic spin-noise, predicted by Bloch in 1946 15 as a weak residual from statistically incomplete cancellation of magnetic fluctuations, was first observed by Sleator et al. in 1985 16 at liquid helium temperature. One can nowadays easily observe nuclear spin-noise on conventional NMR spectrometers at room temperature using cryogenically cooled probes for a large number (∼10^20^−10^22^) of proton spins 2,14, and ^13^C spin-noise 17 as well as heteronuclear 2D NMR with spin noise detection 18 were also recently demonstrated. Even two-dimensional spin-noise spectra were acquired recently from a macroscopic sample.

Spin-noise has been described as a spontaneous emission process, enhanced by the presence of a tuned circuit 19. The exact nature by which the spontaneously emitted energy is transferred to the circuit is less clear. This process was discussed by Hoult and Bhakar 20, where the concept of virtual photons was invoked in order to describe the transfer mechanism. Although potentially of fundamental appeal, this treatment does not lend itself to a quantitative description. In the present context, we focus on the observables related to spin-noise and absorbed circuit noise and do not attempt to address new fundamental insights on the mechanism.

### Noise Line Shape

**Figure 1:**
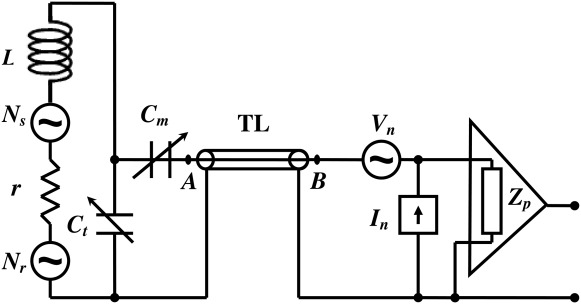
Electronic model of the receiving coil and a preamplifier connected with a transmission line (TL). There are two noise voltage sources, one associated with the resistance *r* of the coil (*N*_r_) and the other associated with the spin-noise from the sample (*N*_s_). *C*_t_ and *C*_m_ are the tuning and matching capacitors, respectively, and *L* is the inductance of the coil. The preamplifier has both voltage and current noise sources *V*_n_ and *I*_n_ respectively, where *Z*_p_ is the preamplifier input impedance.

The Johnson–Nyquist noise expression 21 provides an opportunity for obtaining quantitative agreement with experiments, and can be used to derive the line shapes of the spin-noise signals. The presence of the sample in the circuit (Fig. 1) changes the self-inductance and resistance of the coil, and hence can be modeled via an additional frequency-dependent resistance and inductance within the circuit. For a tuned circuit, Sleator et al. 16,19 and McCoy and Ernst 21 have derived the total spin-noise power *W*(ω) across the terminals of the tuning capacitor from the voltage divider theorem as1
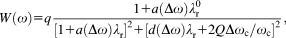
where *Δ*ω is the resonance offset,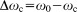 is the tuning offset between the Larmor frequency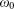 and the nominal tank circuit resonance frequency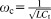 (*L* and *C*_t_ are defined in Fig. 1),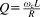 is the quality factor of the circuit, terms not relevant to the line shape are lumped into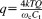, and2
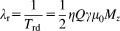
is the radiation damping rate 13. Here *M_z_* is the *z*-magnetization, η the filling factor, and μ_0_ the permeability of free space. The difference between sample temperature *T*_s_ and circuit temperature *T*_c_ is taken into account via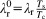, so that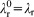 when the spin and coil temperatures are the same (e.g., in an ambient temperature probe) 2.

The absorptive *a*(Δω) and dispersive *d*(*Δ*ω) spectral components3
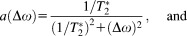
4
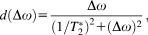
define the line shapes of the NMR noise power signal, which may yield either a “bump” signal or a “dip” signal 14,21 relative to the circuit thermal noise level or various mixed line shapes consisting of absorptive and dispersive contributions. Dips into the thermal noise baseline describe situations in which the spins absorb power from the circuit.

According to the treatment of Eqs. 1–4, a symmetrical “dip” would be observed at the circuit's resonance frequency (i.e., when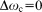). In practice, however, this ideal line shape was observed at considerable tuning offsets from the optimum determined by the conventional tuning procedure (CTO), where the reflection coefficient is minimized, for the majority of probes 2,7,22. The tuning offset at which one observes the symmetric spin-noise or absorbed circuit noise line shape has previously been called SNTO 2, and it varies considerably for different preamplifier-probe combinations. Typical offsets between CTO and SNTO were found to range over hundreds of kHz. A detailed line shape analysis under a variety of conditions can be found elsewhere, including the case of cryogenically cooled probes 2 and hyperpolarized spins 11.

While the treatment leading to Eq. 1 is appealing for its simplicity and for ease of line shape analysis, it does not account for the significant differences between the CTO and SNTO settings. This discrepancy can be traced to the fact that the derivation of Eq. 1 uses the approximation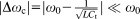. In practice, this condition is often not satisfied. For example, for *Q* = 400, *L* = 40 nH, tuning to
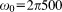
MHz, and series matching 23 to 50  Ω, one obtains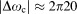 MHz. Under these conditions one can only achieve a symmetric noise line shape if the circuit is detuned by a frequency of that same order of magnitude. Also, in practice, a transmission line cable is connected between the probe circuit and the preamplifier, which further transforms the noise voltage expressions such that the SNTO tuning offset can be altered, as discussed below.

An analytical treatment of the full circuit as modeled in Fig. 1 quickly becomes complicated, but a numerical analysis can be performed easily using the following steps:

1. Calculate the circuit impedance *Z*_A_ at point A.
2. Consider the impedance transformation by the transmission line at point B.
3. Calculate the total noise voltage spectral density at the preamplifier input, including both the Nyquist noise contributions from the sample and the circuit, as well as the preamplifier noise sources.
4. Determine the voltage conversion at the preamplifier input of an emf induced in the circuit and calculate the SNR.
5. The effect of radiation damping and accompanying induced frequency shifts are calculated by determining the relative phase and the amplitude of the current that can flow through the sample coil. For this purpose, it is convenient to calculate the combined impedance of the circuit in series with the sample coil (including the preamplifier input impedance, and its transformation via the transmission line).

### Noise Analysis (Steps 1–3)

The impedance at point A (after series matching) in the circuit of Fig. 1 can be written as5
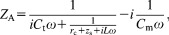
where the contribution to the impedance due to the nuclear spins can be written as6
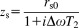
for a Lorentzian resonance line shape with an impedance amplitude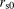 24. Classically, this term can be considered to arise from the Brownian motion of the rotating frame magnetization 24. Equivalently, we could express the spin contribution via the real and imaginary susceptibilities as resistive and inductive elements in the circuit 19. Note that the term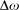 is the offset frequency from the Larmor frequency of the resonance in this case. The frequency-independent (or broad-band) contributions of the sample to the circuit resistance and inductance can be lumped into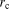 and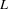 for simplicity.

We note here that by taking the real part of Eq. 5 and using the Nyquist expression for the noise, one can obtain the equivalent of Eq. 1 if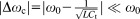 is assumed.

Next, the transmission line is taken into account. The frequency-dependent impedance seen at the preamplifier (point B in Fig. 1) is then 237
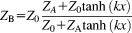
via the standard impedance transformation expression for a lossy transmission line of length *x* (in units of wavelength). A characteristic impedance of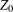 (50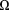 in our case), and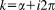 are used, where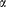 is the loss factor per unit wavelength (for the RG223 cable used in the experiments, a typical value is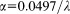 at
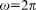
500 MHz). At the small multiples of the wavelength employed, the assumption of zero-loss cables would lead to a negligible error.

At this point the circuit noise can be modeled as a combined noise source for the circuit and spin-noise given by the voltage spectral density8
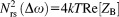
with *Z*_B_ and *Z*_p_ (the preamplifier input impedance) appearing in series.

For the signal-to-noise estimation treatment we follow Refs. 20,25 with slight modifications allowing for complex preamplifier impedance and complex *Z*_B_. The preamplifier noise sources (in addition to a noise-less input impedance *Z*_p_) may be modeled 26 using a current noise source *I*_n_ with a mean square voltage noise of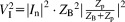 and a voltage noise source *V*_n_ as shown in Fig. 1 20. Even though in practice these two noise sources could be correlated, we neglect this for the current treatment. The effects of this correlation would be negligible in most cases. One can then estimate the total mean square noise voltage at the preamplifier input (across *Z*_p_) as9
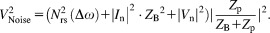


This expression is equivalent to Eq. [A19] of Ref.^20^, except that it also allows for a complex transformed circuit impedance *Z*_B_ and a complex preamplifier impedance *Z*_p_. It will be shown below that optimal SNR is achieved when *Z*_B_ is real.

The spectrum of the combined noise from the coil resistance, the nuclear spins, and the pre-amplifier can be simulated based on Eq. 9 and plotted vs.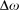. The preamplifier noise sources, as modeled here, add frequency-independent noise. For line shape analysis, one can neglect these contributions (but not for SNR calculations). Equations 7–9 predict a shifting of the SNTO position in a periodic fashion as a function of the line length *x* as will be shown below.

### Signal-to-Noise analysis (Step 4)

Following a pulse with flip angle θ, let the amplitude of the emf induced in the receiving coil by the precessing nuclear magnetization be ξ. Based on energy conservation, this voltage will be transformed to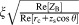 at the input of the preamplifier. Usually, one would like to determine the SNR of a small signal away from the signal of bulk solvent. In this case one can safely assume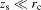 and neglect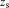. We therefore drop the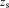 term, and the voltage at the preamplifier input from this induced signal becomes10
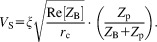


The SNR is then given by dividing *V*_S_ by *V*_Noise_ (Eqs. 8–10),11
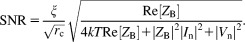


It is remarkable at this point that the preamplifier input impedance, *Z*_p_ drops out of the SNR calculation. Equation 11 shows that SNR is largest when *Im*[*Z*_B_] = 0, and differentiating with respect to *Re*[*Z*_B_] and solving for *Re*[*Z*_B_] gives the optimum impedance at point B as 25,2612
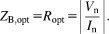


If the circuit (including the transmission line) is matched to this optimum impedance, the best available SNR is13
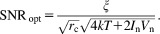


It is readily seen that the condition for optimal SNR (Eq. 12) depends on the noise properties of the preamplifier rather than optimal matching (to 50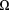).

Changing the transmission line cable length can perform an impedance transformation according to Eq. 7, so that optimal SNR may be achieved. A correlation between the preamplifier noise sources *I*_n_ and *V*_n_ can also be considered, but has little effect on *Z*_opt_ 25. Equation 13 also shows that the presence of the preamplifier degrades the SNR by a factor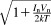 (preamplifier noise factor) 20.

### Radiation Damping and Resonance Frequency Shifts (Step 5)

The noise and pulsed signals further show marked frequency shifts, arising from an effect known as frequency pulling, which can be explained by radiation-damping induced frequency shifts 27. Others have reported that such frequency shifts depended on mistuning 1, and it seemed natural to draw this connection here. Following Ref.^27^, one can trace the appearance of resonance frequency shifts to non-canceled reactive impedances in the circuit. To develop this viewpoint here, it is convenient to consider the preamplifier impedance as transformed via the transmission line at point A,14
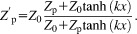


This impedance appears in series with *Z*_A_, hence the total series impedance of the circuit becomes15
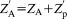


The phase angle ψ of this impedance as defined via16
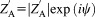
depends of course on the tuning condition, the preamplifier impedance, and the transmission line length. A non-zero phase angle alters the angle between the magnetization vector and the radiation damping back-action field (normally at 90°). Both amplitude and phase have a bearing on radiation damping, and using Eq. 2 it can be shown that the radiation damping time constant 27 is17
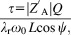
where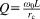 as usual. We will distinguish this quality factor from the experimentally assessed quality factor *Q*_exptl_, as measured in the assembly with the transmission line, which will be shown to vary with cable length.

As a result of the nonzero phase ψ, the current in the coil also leads (cf. Eq. 17 of Ref.^27^) to a resonance frequency shift of18
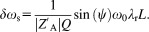


For an idealized circuit where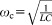, and ψ = 0 at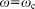, one may further simplify the expressions and obtain the frequency shift in Hz as 119
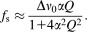
where: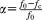. The term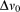 is the resonance line width at half height when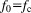,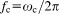, and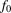 is the resonance frequency of the protons when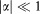.

For the typical case where a transmission line is connected to the resonant circuit, and the preamplifier impedance deviates from the characteristic impedance, however, one needs to consider Eqs. 14–17, where it is seen that a change in cable length will produce changes in the reactance of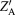, and thereby shifts in the resonance frequencies. Experiments showing these effects are described below.

## MATERIALS AND METHODS

All experiments were recorded on a Bruker Avance 500 MHz spectrometer (11.7 T) equipped with a 5 mm high resolution triple resonance (TXI, H, C, N) ambient temperature probe (sample and circuit temperature 298.3 K) using a H_2_O/D_2_O (9/1) sample. The preamplifier used was a HPPR/2 ^1^H LNA.

NMR noise experiments were performed while the rf-pulse amplifier input cable was disconnected from the ^1^H-preamplifier and terminated, in order to minimize the impact of electronic noise generated by the pulse amplifier and other spectrometer hardware. The receiver gain value (Bruker RG command) was 14,596.5.

Spin-noise data were collected using a pseudo 2D acquisition sequence, acquiring one block of noise per row with a spectral width of 10 ppm. A total of 512 blocks were collected in this way. Each block was Fourier transformed individually to a complex-valued (phase sensitive) spectrum, which was converted to a power spectrum (accumulating the phase sensitive data would lead to a cancellation of the noise signal) and then the rows were summed up to produce a one-dimensional noise spectrum 21.

For the measurements of signal shifts and sensitivity, the pulse durations were optimized so that delivered rf power and flip angles remain the same for all experiments. The pulse durations ranged from 7 μs to 21 μs for 90° pulses and 0.4 μs to 0.6 μs for 5°. The receiver gain (RG) value was 8.

For sensitivity measurements, a single pulse experiment was performed on a 10% ethylbenzene in acetone *d*_6_ sample. The SNR was measured on the quartet signal around 2.74 ppm over a noise range of 0.4 ppm between 4.07 ppm and 4.47 ppm.

All coaxial cables were RG223/U, 50 Ω, manufactured by Pasternack Enterprises, Inc., Irvine, CA. The connectors were female/male 50 Ω RF coax cable BNC connectors, manufactured by Amphenol, Wallingford, CT. The connectors were crimped to the coax cables after cutting to the desired lengths. Cables of different lengths were made in the range of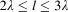 in increments of 0.1λ, where the calculated wavelength was λ = 39.54 cm. The cable length was measured from the rf in/out connector of the preamplifier to the probe.

## RESULTS AND DISCUSSION

**Figure 2:**
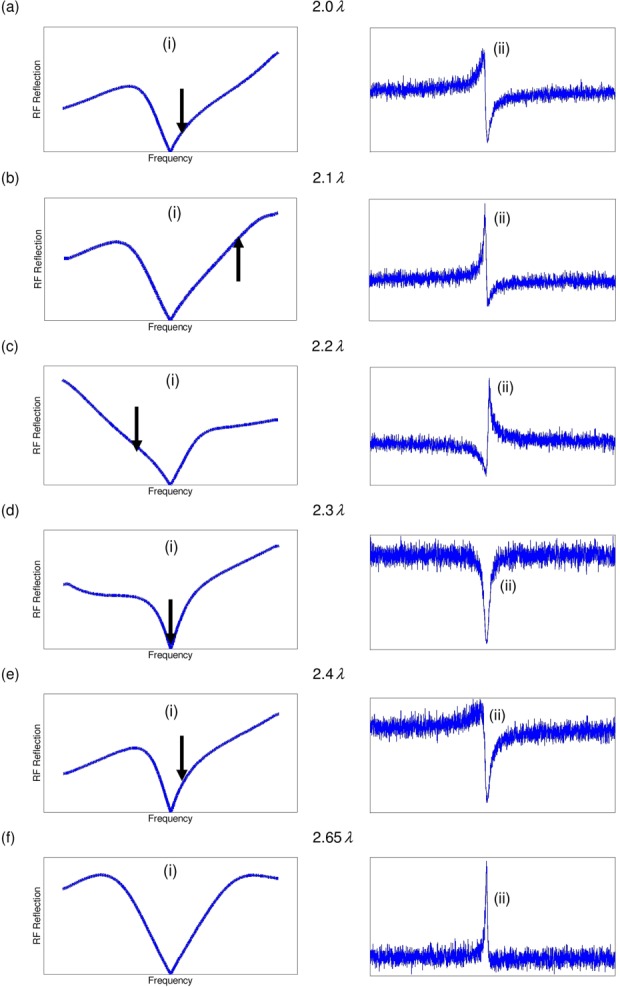
Comparison of tuning curves and spin-noise signals at 500 MHz using the indicated coaxial cable lengths between the preamplifier and the probe. (i) Tuning curves tuned and matched to the CTO at 500.202 MHz. The arrows indicate the SNTO position. (ii) Corresponding spin-noise signals at CTO. (a) 2.0*λ* cable length, SNTO at 500.907 MHz (+705 kHz shift from CTO), (b) 2.1*λ* cable length, SNTO at 502.400 MHz (+2198 kHz shift from CTO), (c) 2.2*λ* cable length, SNTO at 498.837 MHz (−1365 kHz shift from CTO), (d) 2.3*λ* cable length, SNTO at 500.202 MHz (0 kHz shift from CTO), (e) 2.4*λ* cable length, SNTO at 500.594 MHz (+392 kHz shift from CTO), (f) 2.65*λ* cable length, no SNTO found.

Figure 2 shows the tuning curves generated by the spectrometer (Bruker “wobb” command as implemented in Topspin 1.3 on a Bruker AV spectrometer), which represent plots of the difference in voltage drop between an ideal 50 Ω load and the circuit load versus frequency. The cable length has a marked influence on the appearance of the tuning curves. As in previous investigations 2, it is found that the tuning curves rarely assume an ideal symmetrical “dip” form. A steadily increasing lobe on one side can be seen in different situations (either toward increasing or decreasing frequency values). For example, as can be seen in Fig. 2, at cable lengths of 2λ and 2.1λ, the baseline of the tuning curve increases toward higher frequencies and forms a shoulder below the tuning frequency. By contrast, moving to 2.2λ, the baseline of the tuning curve decreases toward higher frequencies and forms a shoulder above the tuning frequency. This behavior repeats at every λ/2 of cable length, as expected.

The SNTO position determined from the noise measurements is based on finding the tuning condition which achieves a symmetric dip line shape (by adjusting tuning and matching). It is seen that the SNTO is always found on the side of the increasing lobe in the tuning curve. In Fig. 2 one can also see the NMR spin-noise signals for each cable length at the CTO. It is found that the negative excursion of the signal occurs at higher frequencies when the SNTO is found at lower frequencies, and vice versa (note that by usual convention, frequency decreases from left to right in the spectra, but for tuning curves typically increases from left to right as shown here).

Between the cable lengths of 2.1λ and 2.2λ, a transition occurs, rather abruptly, and a symmetric tuning curve can be obtained [Fig. 2(f)]. At this cable length, the SNTO position cannot be determined from noise measurements. In previous studies 11, such a situation has also been observed with several probe/preamplifier combinations. In Fig. 2(f), a tuning curve using the same cable length is shown. The tuning curve is symmetric in this regime. In addition, the spin-noise signal at the CTO appears to be close to a perfect bump. This effect can be explained intuitively in combination with insights about radiation damping factors, as described below.

**Figure 3:**
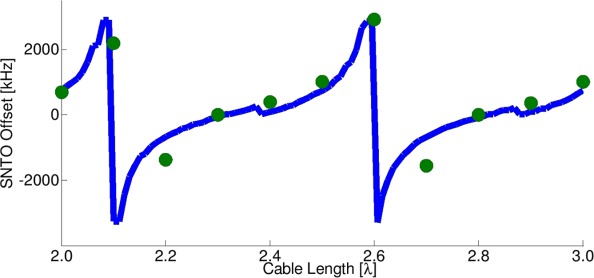
SNTO offset from CTO as a function of coaxial cable length in units of wavelength. Green circles: Measured values, where the SNTO positions were found by determining the tuning frequency that gave a symmetrical dip of the water proton spin-noise signal. Blue line: Simulated curve, values of *L* = 40 nH, *Q* = 400 were used in the simulation. Additional simulation parameters are described in the text.

Figure 3 shows the dependence of the SNTO position on the cable length. It is seen that by changing the cable length, one may reach a regime in which both SNTO and CTO coincide (in this case at 2.3λ and 2.8λ), as also suggested in Ref.^6^. Abrupt changes are seen at approximately 2.1λ and 2.6λ, where the SNTO offset changes from a large positive to a large negative offset. Simulations based on Eqs. 4–8 were then performed in MATLAB using the parameters *Q* = 400 (a typical measured value, see below), *L* = 40 nH, and
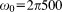
MHz. The circuit resistance *r* was obtained via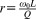 as 0.314 Ω. In addition,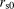 (Eq. 6), the parameter quantifying the spin-noise resistance in relationship to *r* was determined as 0.266 Ω from Fig. 2(f ii), where a preamplifier noise figure of 1.1 was assumed. Using a spectrum analyzer to determine the preamplifier impedance *Z*_p_ gave values in the range of 70–80 Ω with approximately 30 Ω reactive contributions (measured at 20 dBm). These measurements are likely incorrect because the power used by the analyzer probably saturated the preamplifier. Also, values of 500 Ω are much more common for NMR spectrometer preamplifiers and the simulations produced a much better fit with this value. An additional transmission line length of 0.4λ had to be added in the simulation to bring the simulated and experimental curves into agreement. It is easy to rationalize that such an additional line length could account for the internal electrical line lengths within the probe assembly and the preamplifier module.

The results of the simulation are shown by the solid blue line in Fig. 3 which fit well the experimentally observed trends. Some ripples are seen in the simulated curve, likely as a result of instabilities in the minimization algorithm (MATLAB “fminsearch” function) that was used to find the SNTO condition.

**Figure 4:**
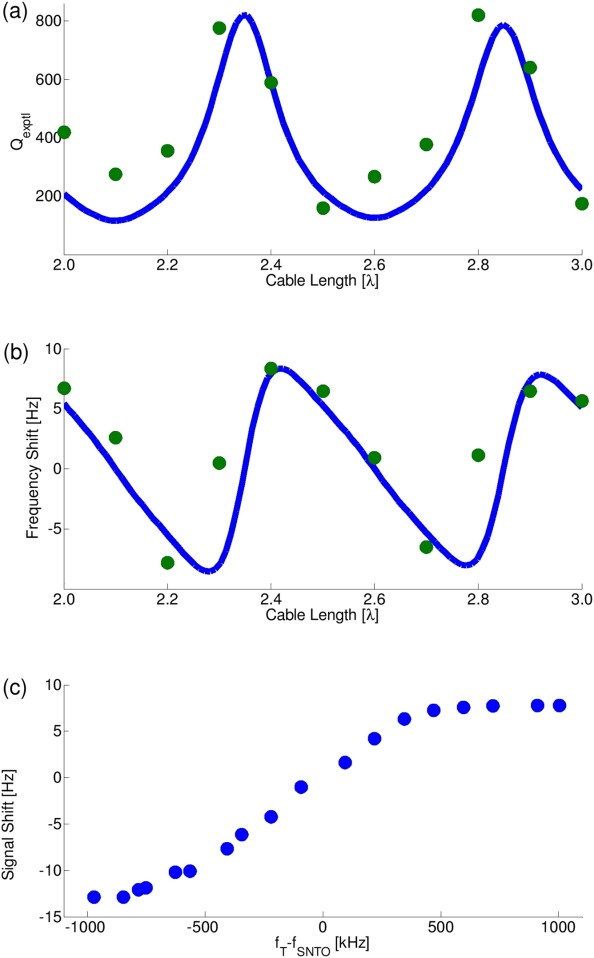
(a) *Q*_exptl_ as a function of cable length in units of wavelength. Green circles: Measured *Q*_exptl_. *Q*_exptl_ was calculated using the tuning frequency (all were done at CTO frequency), divided by the width of the tuning curve at half height from the baseline. Blue line: Simulated *Q*_exptl._ (b) Resonance frequency shift at CTO as a function of coaxial cable length in units of wavelength. Green circles: Measured values. Blue line: Simulation. (c) Frequency shifts of water proton noise signals using a 2.0λ coax cable length. The frequency shift is plotted as a function of tuning offset *f*_T_ − *f*_SNTO_ for values in the range of −1,000 kHz < *f*_T_ − *f*_SNTO_ < 1,000 kHz.

Figure 4(a) shows the experimentally determined “quality factor” *Q*_exptl_ as calculated by dividing the resonance frequency by the width of the tuning curve at half height (the tuning curve is represented in terms of voltage on the spectrometer). This parameter is measured as a function of transmission line length (when tuning to the CTO frequency). The maxima of this curve show the configurations at which the radiation damping effects are strongest. The simulated curve is represented as a solid blue line and is based on calculating 1/τ from Eq. 17. The vertical scaling of this simulated curve is treated as an adjustable parameter due to the many empirical constants that enter this equation. The shape of the curve, however, clearly follows the experimental curve, displaying both maxima and minima of *Q*_exptl_. This effect also illustrates that it is inherently unreliable to assess *Q* factors via reflection coefficient measurements.

The symmetric spin-noise line shape of Fig. 2(f, ii) was obtained in a regime where radiation damping was lowest, thereby indicating that the overall resistance in the network was maximal. One can then explain the appearance of a “bump” spin-noise line shape as follows: From the Nyquist relation (Eq. 9) one obtains the voltage spectral density, which includes both the circuit and the spin contribution to the resistance. In order to obtain current, however, one divides by the absolute square of the total inductance, in which the contribution of the spin-noise becomes minimal. Hence the numerator in this expression becomes dominant (consisting of the sum of *N*_r_ and *N*_s_), which leads to the appearance of a “bump” signal.

As outlined above, and described previously 2,27,28, strong radiation damping should lead to large frequency shifts of the signals. In our case, we have seen shifts spanning up to ∼20 Hz. Figure 4(b) shows the frequency shifts of the resonance lines taken at all the sampled cable length positions, and shows a comparison with a calculated frequency shift curve using Eq. 18. As with *Q*_exptl_, here the vertical scaling of the simulated curve was taken as an adjustable scaling factor, since a number of experimental parameters enter the equation, which are difficult to determine independently. A good correlation with the trend in *Q*_exptl_ values is found, thus illustrating the link between *Q*_exptl_ and radiation-damping-induced shifts. Notably, the largest shifts are found where *Q*_exptl_ is maximal, but in this region, they also switch from large positive to large negative shifts. Zero shifts could therefore also be found in this region by carefully adjusting the cable length but would not be stable. Alternatively, zero shifts can also be found at the minima of *Q*_exptl_, as one would expect. At *Q*_exptl_ maxima, it is seen by comparison with Fig. 3 that the SNTO can be made to coincide with the CTO, while at *Q*_exptl_ minima, the SNTO cannot be determined as it switches from a large positive to a large negative offset.

For a given line length, the frequency shift can be changed by tuning and matching off-resonance. Figure 4(c) shows the range of frequency shifts that can be observed in this way at a line length of 2.4λ, where *Q*_exptl_ is approximately maximal. The experimental points are also compared with the simulated curve based on Eq. 19 and a good agreement can be found. This behavior is similar to what was observed in earlier studies 1,28.

**Figure 5:**
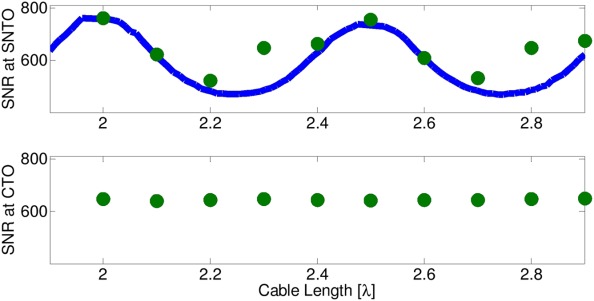
SNR values as a function of cable length in units of wavelength. Green circles: Measured SNR values at (a) SNTO and (b) CTO. Blue line: Simulated SNR values (for SNTO tuning plot in (a) only). The simulated plot is shifted by −0.1λ to obtain the best fit as described in the text.

The SNR was assessed by performing one-dimensional pulse spectra using a sample consisting of 10% ethylbenzene in acetone *d*_6_. The SNR values were recorded for cable lengths from 2λ to 2.9λ (representing a full cycle) at both the SNTO and CTO settings (Fig. 5). The SNTO for an acetone sample showed the same trend as the one for a water sample. Larger differences were encountered with up to 200 kHz difference compared to SNTO for a water solution for the SNTO maxima and minima. It was found that the SNR fluctuated somewhat for the CTO setting [Fig. 5(b)], but did not show pronounced maxima or minima. This behavior is indeed expected if the circuit is tuned and matched very close to 50 Ω. The fluctuations there indicate that the circuit cannot be matched exactly to 50 Ω. At the SNTO, the pulses were recalibrated for each cable length. It was found here that the largest SNR appeared for cases where the SNTO was at a tuning frequency of approximately 800 kHz higher than the one for CTO. The solid blue line in Fig. 5(a) shows the simulated SNR curve according to Eq. 11 with the vertical scale being an adjustable parameter. It was found that the simulated SNR curve was offset by −0.1λ from the maxima and minima of the experimental curve. The origin of this offset in cable lengths is not known at present. Changes in *Z*_opt_ of both the real and imaginary parts would not explain such a shift as can be verified from Eq. 10. However, the appearance of one maximum and one minimum in the SNR curve per half wavelength is well understood based on this treatment.

The main result obtained from Fig. 5 is, thus, that the positions of optimal sensitivity and SNTO (“ideal spin-noise dip”) depend on cable lengths and only coincide with certain cable lengths. This feature explains previous findings, where SNTO and SNR optimum were seen to differ between different probe-cable-preamplifier combinations.

The maximum SNR is found approximately where *Q*_exptl_ is close to its maximum value, hence under conditions of maximum radiation damping. Given the very different derivations of SNR and *Q*_exptl_, however, one can say that this finding is rather coincidental. The position of optimal SNR may further be affected by a change in *Z*_opt_ via the noise sources *I*_n_ and *V*_n_, as was pointed out previously 20,25. It would be desirable for many applications to find settings where the SNR would be maximal and the radiation damping minimal (minimal *Q*_exptl_). Such modifications could in theory be performed irrespective of the preamplifier input impedance *Z*_p_, but in practice, there are limits to the range of such adjustments. The use of additional impedance transformation circuits within, before, or after the transmission line, which would operate asymmetrically in the forward and reverse directions 6, could offer additional flexibility.

## CONCLUSIONS

We have discussed here the influence of the coaxial cable length between the preamplifier and the probe on a number of parameters, such as experimentally observable quality factors, sensitivity, radiation-damping-induced frequency shifts, and the noise (absorbed circuit noise and spin-noise) line shapes. It is described how changing the cable length allows one to find a tuning regime where the maximum SNR is achieved, while the optimal transmission setting has a different optimum. Further, it is shown that one can also make the optimal transmission and optimal noise reception settings coincide. The spin-noise and absorbed circuit noise spectral line shapes have a marked dependence on the tuning curves, and at certain cable lengths, the perfect SNTO line shape cannot be found. Radiation-damping-induced frequency shifts are seen to correlate with the cable lengths in a similar fashion as the experimentally observed quality factor values do. The SNTO is not always located where the frequency shift is minimal, nor is it always indicative of SNR optima. Most of these effects can be explained by the coaxial cable acting as a two-way impedance transformer, which matches the impedances at both the probe circuit, as well as, the preamplifier. The practical considerations described herein are useful for the optimization of different spectrometer setups for radiation damping blocking, transmission, or reception, or all combined. On commercial cryogenically cooled probes, fewer options are available for optimization since the cold part of the preamplifier is rigidly connected to the probe.
